# MEDIASTinal staging of non-small cell lung cancer by endobronchial and endoscopic ultrasonography with or without additional surgical mediastinoscopy (MEDIASTrial): study protocol of a multicenter randomised controlled trial

**DOI:** 10.1186/s12893-018-0359-6

**Published:** 2018-05-18

**Authors:** Jelle E. Bousema, Marcel G. W. Dijkgraaf, Nicole E. Papen-Botterhuis, Hermien W. Schreurs, Jos G. Maessen, Erik H. van der Heijden, Willem H. Steup, Jerry Braun, Valentin J. J. M. Noyez, Fieke Hoeijmakers, Naomi Beck, Martijn van Dorp, Niels J. M. Claessens, Birgitta I. Hiddinga, Johannes M. A. Daniels, David J. Heineman, Harmen R. Zandbergen, Ad F. T. M. Verhagen, Paul E. van Schil, Jouke T. Annema, Frank J. C. van den Broek, MEDIASTrial study group, Maggy Youssef-El Soud, Wim J. van Boven, Thirza Horn, Pepijn Brocken, Rajan R. S. Ramai, Nicole P. Barlo, Anne-Marie C. Dingemans, Jan-Willem Lardenoije, Anthonie J. van der Wekken, Caroline van de Wauwer, Robert Th. J. Kortekaas, Wessel E. Hanselaar, Herman Rijna, Martin P. Bard, Femke H. M. van Vollenhoven, Gabi B. Murrmann, Gerben P. Bootsma, Yvonne Vissers, Eelco J. Veen, Cor H. van der Leest, Emanuel Citgez, Eino B. van Duyn, Geertruid M. H. Marres, Eric R. van Thiel, Xiang H. Zhang, Wout B. Barendregt, Julius P. Janssen, Niels Smakman, Femke van der Meer, Mohammed D. Saboerali

**Affiliations:** 1 Department of Surgery, Máxima Medical Center, PO BOX 7777, 5500 MB Veldhoven, the Netherlands; 2 University of Amsterdam, Amsterdam, the Netherlands; 3 Clinical Research Unit, Academic Medical Center, Amsterdam, the Netherlands; 4 Department of Surgery, Noordwest Ziekenhuisgroep, Alkmaar, the Netherlands; 5 Department of Cardiothoracic Surgery, Maastricht University Medical Center, Maastricht, the Netherlands; 6 Department of Pulmonary Medicine, Radboud University Medical Center, Nijmegen, the Netherlands; 7 Department of Surgery, HagaZiekenhuis, Den Haag, the Netherlands; 8 Department of Cardiothoracic Surgery, Leiden University Medical Center, Leiden, the Netherlands; 9 Department of Surgery, KU Leuven, Leuven, Belgium; 10 Department of Surgery, Leiden University Medical Center, Leiden, the Netherlands; 11 Dutch Institute for Clinical Auditing, Leiden, the Netherlands; 12 Department of Pulmonary Medicine, Rijnstate ziekenhuis, Arnhem, the Netherlands; 13 Department of Pulmonary Medicine, University of Groningen and University Medical Centre Groningen, Groningen, the Netherlands; 14 Department of Pulmonary Medicine, VU University Medical Center, Amsterdam, the Netherlands; 15 Department of Surgery, VU University Medical Center, Amsterdam, the Netherlands; 16 Department of Cardiothoracic Surgery, VU University Medical Center, Amsterdam, the Netherlands; 17 Department of Cardiothoracic Surgery, Radboud University Medical Center, Nijmegen, the Netherlands; 18 Department of Thoracic and Vascular Surgery, Antwerp University Hospital, Antwerp, Belgium; 19 Department of Pulmonary Medicine, Academic Medical Center, Amsterdam, the Netherlands

**Keywords:** Mediastinal staging, Mediastinoscopy, Non-small cell lung carcinoma, Endosonography, Thoracic surgery

## Abstract

**Background:**

In case of suspicious lymph nodes on computed tomography (CT) or fluorodeoxyglucose positron emission tomography (FDG-PET), advanced tumour size or central tumour location in patients with suspected non-small cell lung cancer (NSCLC), Dutch and European guidelines recommend mediastinal staging by endosonography (endobronchial ultrasound (EBUS) and endoscopic ultrasound (EUS)) with sampling of mediastinal lymph nodes. If biopsy results from endosonography turn out negative, additional surgical staging of the mediastinum by mediastinoscopy is advised to prevent unnecessary lung resection due to false negative endosonography findings. We hypothesize that omitting mediastinoscopy after negative endosonography in mediastinal staging of NSCLC does not result in an unacceptable percentage of unforeseen N2 disease at surgical resection. In addition, omitting mediastinoscopy comprises no extra waiting time until definite surgery, omits one extra general anaesthesia and hospital admission, and may be associated with lower morbidity and comparable survival. Therefore, this strategy may reduce health care costs and increase quality of life. The aim of this study is to compare the cost-effectiveness and cost-utility of mediastinal staging strategies including and excluding mediastinoscopy.

**Methods/design:**

This study is a multicenter parallel randomized non-inferiority trial comparing two diagnostic strategies (with or without mediastinoscopy) for mediastinal staging in 360 patients with suspected resectable NSCLC. Patients are eligible for inclusion when they underwent systematic endosonography to evaluate mediastinal lymph nodes including tissue sampling with negative endosonography results. Patients will not be eligible for inclusion when PET/CT demonstrates ‘bulky N2-N3’ disease or the combination of a highly suspicious as well as irresectable mediastinal lymph node. Primary outcome measure for non-inferiority is the proportion of patients with unforeseen N2 disease at surgery. Secondary outcome measures are hospitalization, morbidity, overall 2-year survival, quality of life, cost-effectiveness and cost-utility. Patients will be followed up 2 years after start of treatment.

**Discussion:**

Results of the MEDIASTrial will have immediate impact on national and international guidelines, which are accessible to public, possibly reducing mediastinoscopy as a commonly performed invasive procedure for NSCLC staging and diminishing variation in clinical practice.

**Trial registration:**

The trial is registered at the Netherlands Trial Register on July 6th, 2017 (NTR 6528).

**Electronic supplementary material:**

The online version of this article (10.1186/s12893-018-0359-6) contains supplementary material, which is available to authorized users.

## Background

Lung cancer is a common disease with over 12,000 new Dutch cases annually and 1.8 million worldwide. In the Netherlands 9175 new non-small cell lung cancer (NSCLC) patients were diagnosed in 2017 [1, 2]. At diagnosis about 80% of patients already have distant or regional metastases, whereas only 20% is eligible for surgical treatment with curative intent. With (the suspicion of) potential curable NSCLC, patients undergo computed tomography (CT) and fluorodeoxyglucose positron emission tomography (FDG-PET) in order to obtain information about locoregional and distant disease. In case of absence of distant metastases but presence of suspicious lymph nodes on PET/CT, Dutch and European guidelines recommend mediastinal staging by endobronchial (EBUS) and/or endoscopic esophageal ultrasonography (EUS) with sampling of suspicious mediastinal lymph nodes [3]. In patients with non-FDG-avid tumour, central tumour location or with peripheral tumours > 3 cm, mediastinal staging is recommended as well. In case of negative biopsy results from endosonography, surgical staging of the mediastinum by mediastinoscopy is advised to prevent possible unnecessary surgery due to false negative endosonography findings. Generally only patients without N2–3 metastases after mediastinoscopy are eligible for intended curative anatomic resection. Patients with pathologically proven N2–3 mediastinal lymph node metastases are usually recommended to undergo first line chemoradiation instead of surgery since no survival benefit has been demonstrated by additional surgery [4]. When mediastinoscopy demonstrates potentially resectable N2 metastases several treatment strategies can be followed: induction chemotherapy followed by surgery, induction chemoradiotherapy followed by surgery or definitive chemoradiotherapy [5, 6].

In a randomized trial comparing endosonography (EBUS and EUS) versus surgical staging, the sensitivity for mediastinal nodal spread was 85% for endosonography and 79% for mediastinoscopy with a total cohort N2–3 prevalence of 46% [7]. Mediastinoscopy diagnosed mediastinal lymph node metastases after negative endosonography in 9.2% of patients, resulting in a combined sensitivity of 94%, which is the rationale of recommending additional mediastinoscopy after negative endosonography. However, to detect one case of single level N2 disease, 11 patients need to undergo additional surgical staging at the expense of morbidity, delay in diagnostic work-up as well as financial costs. Several more non-randomized comparative studies also demonstrated higher sensitivity for endosonography than for mediastinoscopy [8–10]. These studies have raised questions on how to identify false negative endosonography cases in order to significantly reduce or even abandon additional surgical staging.

Moreover, mediastinoscopy is associated with minor (3.2%) and major (3.5%) complications, sporadic mortality (< 1%) and encompasses an additional invasive surgical procedure necessitating general anaesthesia and delaying definite curative treatment [7, 11]. Therefore, significantly reducing or even omitting the need for mediastinoscopy after negative endosonography may reduce morbidity and mortality, as well as costs.

On the other hand, if all patients with negative endosonography results would undergo an anatomic pulmonary resection without additional mediastinoscopy, at least 9.2% of patients would postoperatively turn out to have unforeseen N2 disease. In the ASTER trial, all patients with negative endosonography results and subsequent positive mediastinoscopy had single lymph node station disease and one out of three had micrometastases only [7]. Cerfolio et al. demonstrated good 5-yr survival by surgical resection and adjuvant therapy in single nodal station unforeseen N2 disease (40%) and hereby reached comparable survival as in patients with N1 disease [12]. Several others also showed favourable 5-yr survival rates in these patients [13, 14]. To strengthen these figures, recent survival data from the ASTER trial demonstrated equal 5-yr survival rates of 35% in both randomization groups, despite significantly different detection rates of upfront N2 disease [15]. Therefore, surgical treatment of minimal unforeseen N2 disease instead of definite chemoradiation is increasingly considered as treatment option as well [5, 6]. In addition, the revised European Society of Thoracic Surgery (ESTS) guideline of mediastinal staging states that there is room for trials evaluating surgical treatment instead of chemoradiation for minimal N2 disease [16]. The aim of this study is to compare the cost-effectiveness and cost-utility of mediastinal staging strategies including and excluding mediastinoscopy.

## Methods/design

### Hypothesis

Omitting mediastinoscopy after negative endosonography in mediastinal staging of NSCLC does not result in an unacceptable percentage of unforeseen N2 disease at surgical resection. In addition, omitting mediastinoscopy will shorten time until definitive surgery, will prevent one general anaesthesia and hospital admission and will be associated with lower morbidity and comparable survival. Therefore, this strategy may increase quality of life and reduce health care costs.

### Objective

The main objective of the proposed randomized trial is to compare the cost-effectiveness and cost-utility of mediastinal staging strategies including and excluding mediastinoscopy, provided that non-inferiority of excluding mediastinoscopy regarding unforeseen N2 disease can be demonstrated.

### Study design

**Fig. 1:**
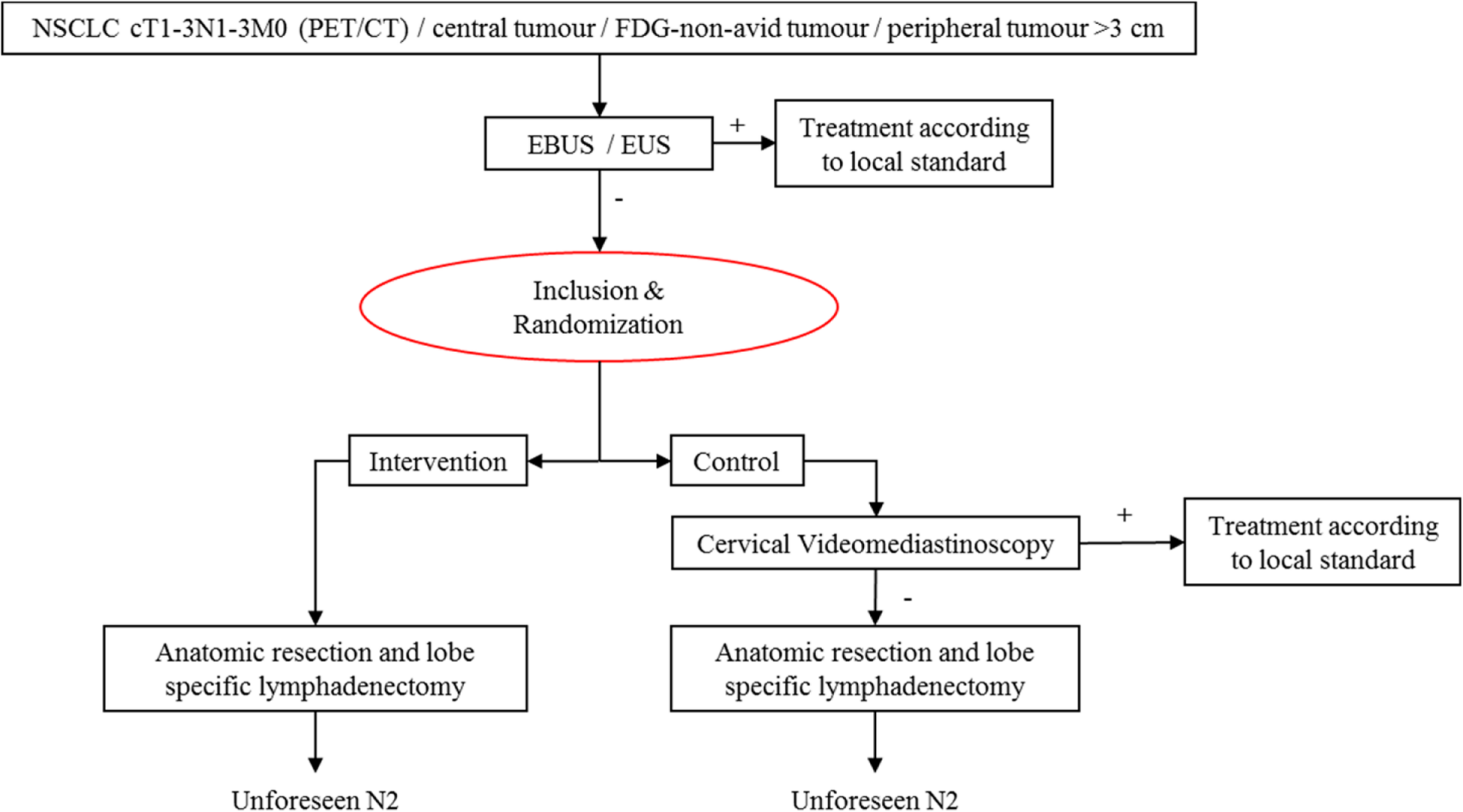
Flowchart

This will be a multicentre parallel randomized trial comparing two diagnostic strategies (with or without mediastinoscopy) for mediastinal staging in patients with suspected NSCLC, based on non-inferiority. The MEDIASTrial flowchart is shown in Fig. 1.

### Randomization

After written informed consent, provided at the outpatient clinic, patient data are entered into a computerized database (Research Manager) and with an unchangeable computer generated number patients will be randomized (1:1) to undergo either mediastinal staging *with* or *without* additional mediastinoscopy. Randomization will be stratified by type of treatment centre and, for its potential impact on cost-effectiveness outcomes, by age below/above 66 years. Variable block size randomization will be applied.

### Blinding

Blinding of patients and physicians during staging and treatment is unfeasible, since the two diagnostic strategies and dependent treatments are highly different in nature and in associated care.

### Study population

Patients are eligible for inclusion in this trial when they meet the following eligibility criteria:

1. Diagnosed (with pathological proof by bronchoscopic or transthoracic biopsy) or suspected (based on CT and FDG-PET) with NSCLC.
2. CT and FDG-PET scan have excluded distant metastasis (stage IV disease) or an irresectable primary tumour (judged by thoracic surgeon, based on imaging).
3. One of the criteria defining the need for mediastinal staging are met according to the European and Dutch guidelines [16, 17]:

- PET/CT of the chest demonstrates CT-enlarged (short axis > 1 cm) or FDG-PET avid hilar (cN1) or mediastinal (cN2-N3) lymph nodes. PET is considered positive if the standardized uptake value (SUV) > 2.5, which is the ratio of the regional radioactivity concentration divided by the injected amount of radioactivity normalized to body weight
- CT demonstrates a centrally located primary tumour, which is defined by visibility of the tumour on video bronchoscopy within the main stem bronchi; or tumour proximity to the mediastinum < 0.5 cm on CT; or location of the tumour within the inner 1/3 of the thorax. Whether the tumour fulfils these criteria will be discussed by the local multidisciplinary meetings
- FDG-PET demonstrates a FDG non avid primary tumour.
- Peripheral lung tumours (outer two third of the chest on CT) larger than 3 cm on CT

### Inclusion criteria

1. Patients underwent systematic EBUS, preferably added by EUS, to evaluate mediastinal lymph nodes including tissue sampling with negative biopsy results. Adequate systematic EBUS / EUS is defined as evaluation of at least lymph node stations 4 L, 7 and 4R by EBUS [18]. Preferably stations 4 L, 7 and 8 should be evaluated by subsequent EUS as well. Lymph nodes in stations 4 L, 7 and 4R larger than 8 mm as well as all CT-enlarged (> 1 cm) and FDG-avid (SUV > 2.5) mediastinal lymph nodes should be sampled by at least 3 needle aspirations. In case of FDG-avid nodes that are smaller than 8 mm and have unsuspicious appearance on endosonography punctures are not obligatory.
2. Patients should be fit enough to undergo resection of the primary tumour by either pneumonectomy, (bi)lobectomy or segmentectomy, judged during the local multidisciplinary meeting. Assessment of fitness includes pulmonary function testing (spirometry and diffusing capacity of the lung for carbon monoxide), followed by cardiopulmonary exercise testing (CPET) if deemed necessary by the multidisciplinary board.
3. Patients should be able to undergo cervical mediastinoscopy (no current tracheostomy or previous mediastinoscopy)
4. Patient age of 18 years or older and able to give informed consent and fill out questionnaires.

### Exclusion criteria

1. PET/CT demonstrates ‘bulky N2–3’ disease. Definition of ‘bulky’ N2–3 disease is copied from the definition given in the revised ESTS guideline: mediastinal infiltration of more than one mediastinal zone where the discrete lymph nodes cannot be distinguished or measured during CT or endosonography; or two or more lymph nodes with a short axis of > 2.5 cm in more than one mediastinal zone (according to the international association for the study of lung cancer (IASLC) node map) [16, 19].
2. The combination of a highly suspicious as well as irresectable mediastinal lymph node. High suspicion of a lymph node is defined as FDG-PET SUV > 5 and at least 3 of the following ultrasonographic malignant criteria: round shape, sharp borders, hypo- echoic texture and short axis > 10 mm. Whether a lymph node is irresectable is judged by the surgeon, based on extracapsular growth or growth into vital structures or due to unreachable location (for example location in lymph node station 4 L in case of a right sided operation).
3. Non-correctable coagulopathy (international normalized ratio > 1.7 or platelet count < 50 × 10^9^/l).
4. Insufficient comprehension of the Dutch language to understand the trial information and to complete the questionnaires during follow-up period.

### Participating centres

Twenty Dutch hospitals and one Belgian hospital participate in the MEDIASTrial study group, including seven academic and fourteen non-academic centres, and will enroll patients.

### Intervention

Patients will undergo immediate anatomic resection of the primary tumour by either pneumonectomy, (bi)lobectomy or segmentectomy according to patient and tumour characteristics as discussed by the local multidisciplinary lung meeting in the participating centre. If possible, patients are treated by video-assisted thoracoscopy (VATS) or robotic-assisted thoracic surgery (RATS). During the surgical procedure, at least a lobe-specific mediastinal lymph node dissection will be done according to European guidelines [3, 20].

### Usual care (comparison)

According to current national and international guidelines, patients will first undergo cervical mediastinoscopy. For this trial, only videomediastinoscopy will be used. This procedure will be done under general anaesthesia, and at least lymph node stations 2R, 4R, 4 L, and 7 should be sampled for right-sided tumours, whereas at least station 4 L, 4R and 7 should be sampled for left-sided tumours. Station 2 L will only be removed when visualized or in case of suspicion based on PET/CT [3, 16].

When histopathological examination of the resected lymph nodes does not demonstrate metastases, patients will undergo additional anatomic lung resection and at least, a lobe-specific lymph node dissection as described under ‘intervention’, which will serve as reference standard in both randomization groups.

When histopathology after mediastinoscopy demonstrates N2–3 metastases, patients are generally recommended to undergo definite chemoradiation. When mediastinoscopy demonstrates potentially resectable N2 metastases several treatment strategies can be followed: surgery and adjuvant chemotherapy, induction chemotherapy followed by surgery, induction chemoradiotherapy followed by surgery or definitive chemoradiotherapy [5, 6]. Discussion within the local multidisciplinary meeting will decide exact treatment in these cases. Differences in treatment between participating centres will be adjusted by stratification per setting (academic, non-academic). These patients will be followed according to the routine follow-up scheme of this study.

### Informed consent procedure

Consecutive patients will be checked for eligibility during the multidisciplinary meetings in the participating centres by the involved physicians (surgeon, pulmonologist, radiation oncologist, radiologist, nuclear medicine physician and pathologist). All patients fulfilling the inclusion criteria will subsequently be informed about the trial by their local pulmonologist or surgeon at the next outpatient clinic visit (depending on local logistics). The MEDIASTrial informed consent form is attached as Additional file 1. After informed consent is given, randomization will take place by a computerized randomization program, using Research Manager Software and patients will be further staged and treated according to the study protocol. Patients unable or refusing to provide informed consent will be treated according to current clinical guidelines, which is additional surgical staging by mediastinoscopy.

### Quality assurance

All participating centres should adhere to the European Association of Nuclear Medicine procedure guidelines of FDG-PET/CT for tumour imaging to guarantee high quality of performing, interpreting and reporting FDG-PET/CT-scan [21].

To assure high quality of endosonography, endoscopists have been trained in EBUS and EUS during their training as pulmonologist. Additionally, endoscopists participating in this study have performed a specific endosonography lung cancer staging training module. Also they have passed an EBUS skill and assessment tool (EBUSAT) evaluating structural EBUS anatomy and standardised mediastinal nodal sampling. The EBUSAT has demonstrated reliable and valid assessment of competence [18]. On individual basis, both EBUS simulator training and clinical EBUS-EUS training will be offered if necessary.

To assure high quality of mediastinoscopy and lymphadenectomy, surgical protocols and demands have been written and will be monitored during the trial.

### Outcome parameters

The following baseline characteristics will be collected; gender, age at time of randomization, height, weight, location of the primary tumour, World Health Organization (WHO) performance state, American Society of Anaesthesiologists (ASA) classification and Tumour, Node, Metastases (TNM) classification (eight edition). Schedule of events is shown in Table 1. To perform the cost-effectiveness and cost-utility analysis, the following primary and secondary outcome measures are chosen:

**Table 1:** Schedule of events

	Enrollment	Baseline	Mediastinoscopy	1 W after mediastinoscopy	Therapy	2 W after start treatment^a^	4 W after start treatment^a^	3 M after start treatment^a^	6 M after start treatment^a^	12 M after start treatment^a^	24 M after start treatment^a^
		T0	T1	T2	T3	T4	T5	T6	T7	T8	T9
Informed consent	X										
Baseline data		X									
eCRF EBUS/EUS		X									
eCRF Mediastinoscopy			X								
eCRF PA Mediastinoscopy				X							
eCRF Therapy					X						
eCRF PA Surgery						X					
eCRF Follow-up^b^				X		X		X	X	X	X
EQ-5D-5 L		+		+		+	+	+	+	+	+
EORTC QLQ-C30		+		+		+	+	+	+	+	+
EORTC QLQ-LC13		+		+		+	+	+	+	+	+
iMTA – iMCQ		+					+	+	+	+	+
iMTA – iPCQ		+					+	+	+	+	+
		Wave 1		Wave 2		Wave 3	Wave 4	Wave 5	Wave 6	Wave 7	Wave 8

^a^Number of weeks or months after start treatment, i.e. surgical partial lung resection, chemotherapy or radiotherapy

^b^eCRF Follow-up contains information about survival, recurrence of disease and serious adverse events

^x^Digital report by local investigator

^+^Digital or written report by patient

### Primary outcome measure (for non-inferiority)

1. As the goal of accurate mediastinal staging is the prevention of performing lung surgery in patients with N2 disease (e.g. unforeseen N2), the proportion of patients with unforeseen N2 disease after final lung resection and mediastinal lymphadenectomy is considered as primary outcome measure for the non-inferiority design of this trial.

### Secondary outcome measures

- The total number of days of hospital care, defined as the total number of days in hospital after randomization during a follow up period of 2 years. Every day in hospital (including outpatient clinic visits and day care treatments) related to NSCLC diagnosis, treatment or follow-up will be counted.
- Costs of mediastinal staging strategies (including or excluding surgical mediastinal staging) from a societal perspective, based on primary data (see also economic evaluation).
- Morbidity: the combination of major morbidity and 30-day mortality is chosen as composite outcome measure. Major morbidity is defined as the proportion of patients having morbidity of grade III-IV (Clavien-Dindo classification) or recurrent laryngeal nerve injury, which is a specific serious adverse event associated with mediastinoscopy [22].
- Overall 2-year survival, defined as the proportion of patients alive at 2 years follow-up, and 2-year disease-free survival, defined as the proportion of patients alive without evidence of relapse of disease at 2 years follow-up. Follow-up is done by pulmonologists at 3 monthly intervals during the first year and 6 monthly intervals during the second year. Hereafter, yearly follow-up will be done until 5 years after treatment. This follow-up scheme is in concordance with the Dutch guideline of NSCLC. Finally, 5-year overall and disease-free survival will be measured after 5 years of follow-up.
- Generic and disease-specific health related quality of life will be measured at baseline, 1 week after mediastinoscopy (only randomization group including mediastinoscopy), 2 weeks after start treatment (e.g. anatomic resection or chemo- and/or radiotherapy), at 4 weeks, at 3 months, at 6 months, at 1 year and at 2 year follow-up by the EQ-5D-5 L, EORTC QLQ-C30 and QLQ-LC13 questionnaires.

### Sample size calculation

In the ASTER trial, surgical staging with mediastinoscopy had a sensitivity of 79% for detecting N2 disease vs. 94% for the combined use of endosonography and mediastinoscopy in a population with 75% PET/CT N2–3 disease positives [7]. Negative histology after staging was followed by surgical mediastinal lymphadenectomy, which provided the best possible reference standard. The difference in sensitivities between the two staging strategies in this trial led to unforeseen N2 rates of 14.3% after surgical staging versus 6.9% after endosonography ánd mediastinoscopy. Despite this difference in diagnostic staging, 5-year survival rates were completely equal (35% vs. 35%) [15]. Therefore, for our trial we assume that the proportion of unforeseen N2 after omitting mediastinoscopy (experimental arm in our trial) may not exceed 14.3% as upper limit of its 95%-confidence interval (non-inferiority) in order to have no negative impact on survival.

We conducted a systematic review about mediastinal staging (unpublished data). Herein we found a proportion of unforeseen N2 of 6.3% after endosonography combined with mediastinoscopy (control group). We found 6.8% unforeseen N2 nodes in patients staged with endosonography alone, without mediastinoscopy. With these results, we calculated to include 171 patients in each randomization group (power 80%; alpha error 0.025). Based on an assumed 5% drop-out rate of patients after randomization, we aim to include a total of 360 patients.

### Ethics

This study will be performed in accordance with the declaration of Helsinki, 64th WMA General Assembly, Fortaleza, Brazil, October 2013 and in accordance with the Medical Research Involving Human Subjects Act (WMO, the Netherlands). The medical ethical committee of the Maxima Medical Center has approved the study protocol (Medical Ethical Committee (MEC) number W17.063). Important protocol modifications will be communicated as soon as possible with the local investigators and the Dutch Trialregister. Prior to randomization, written informed consent will be obtained from all patients.

### Data safety

After written informed consent, patients will be assigned a study number and clinical data will be registered pseudonymous via Research Manager software. Research Manager software is certified by the ‘Information Security Management System 27001’. The key to the code is safeguarded by the local principal investigator. Quality of Life and Health Economics questionnaires will be coordinated by the Netherlands Comprehensive Cancer Organisation (IKNL), having extensive experience in acquiring information on quality of life in cancer patients in general. The gathered data will be collected in the PROFILES registry by IKNL. The PROFILES registry recently obtained the ‘Data Seal of Approval’.

Monitoring will be done by IKNL according to the MEDIASTrial monitoring plan.

All centers will be visited 3 months after inclusion of the third patient. In case centers have high or low inclusion rate or queries in datamanagment, additional monitor visits will be done. Monitoring will take place with specific attention to informed consent, data monitoring and completeness of case record form.

Local data management will be done by IKNL, having extensive experience with management of local data collection. Collection, storage and analysis of data will be done according to the MEDIASTrial data management plan.

No data safety monitoring board will be established, since this is a diagnostic trial of usual care evaluating diagnostic strategies with an expected low complication rate.

Research data can be presented or published in agreement with the principal investigator (FvdB) only. No research data that can be traced to individual persons will be presented or published. The research data will be reported following the CONSORT guidelines.

### Patient safety

The sponsor/coordinating investigator has an insurance which is in accordance with the legal requirements in the Netherlands (article 7 WMO). This insurance provides cover for damage to research subjects through injury or death caused by the study. The insurance applies to the damage that becomes apparent during the study or within 4 years after the end of the study.

The sponsor/coordinating investigator will report the concerning SAEs through the web portal ToetsingOnline to the accredited METC that approved the protocol, within 7 days of first knowledge for SAEs that result in death or are life threatening followed by a period of maximum of 8 days to complete the initial preliminary report. All other SAEs will be reported within a period of maximum 15 days after the sponsor/coordinating investigator has first knowledge of the SAEs.

In case subjects withdraw from study participation, these patients will undergo treatment and follow-up according to local treatment and follow-up protocols. These individuals will be asked for permission to just register their information on actual treatment and regular follow-up, in order to report outcome of withdrawn cases.

### Data-analysis

The number of patients with pathologically proven N2 disease after final lung resection and lobe specific mediastinal lymphadenectomy divided by the total number of patients who underwent lung resection with lobe specific mediastinal lymphadenectomy is considered the proportion of patients with unforeseen N2 (primary outcome measure). These proportions will be compared between the two randomization groups by the Chi square test, based on intention to treat (ITT). Considering that a non-inferiority hypothesis is tested a per protocol analysis (PP) will also be performed. Both, the ITT and the PP analyses should indicate non-inferiority before the diagnostic strategy without mediastinoscopy will be assessed as non-inferior to the strategy with mediastinoscopy. Incongruent results from the ITT and PP analyses will be discussed. No interim analysis is planned.

The total number of days of hospital care will be counted after randomization during a follow up period of 2 years. The mean (or median) number of days plus standard deviation (or interquartile range) will be compared between groups by the Student’s t-test or Mann Whitney U test depending on the distribution (normally of skewed) of data. The number of patients with either major morbidity or death within 30 days from definite surgery divided by the total number of randomized patients is considered as the proportion of patients with either major morbidity or 30-day mortality (composite outcome measure). These proportions will be compared between the two randomization groups by Chi-square testing. The number of patients alive and the number of patients alive without evidence of relapse of disease after 2 years follow-up divided by the total number of randomized patients are considered as overall and disease-free 2 year survival rates. The log rank test will be used to compare the study arms, based on intention to treat. Generic and disease-specific health-related quality of life will be measured by EQ-5D-5 L, EORTC QLQ-C30 and QLQ-LC13 questionnaires and provide continuous variables that will be compared between the randomization groups by generalized linear mixed modelling. All analyses of secondary outcomes will be carried out on an intention-to-treat basis.

### Economic evaluation

The economic evaluation of both mediastinal staging strategies will be performed as a cost-effectiveness analysis as well as a cost-utility analysis from a societal perspective. The primary outcomes for the cost-effectiveness and cost-utility analyses are the costs per patient without unforeseen N2 and the costs per QALY. The costs per patient free of major complications/death and the costs per patient alive after 2 years follow-up will be considered as secondary outcome measures.

The cost-analysis will include health care costs, out-of-pocket expenses and costs of production loss. The direct medical costs will include the costs of all diagnostic procedures, therapeutic (repeat) interventions, medication, admissions, day care treatments, specialist consultations, and out-of-hospital care (like general physician, physiotherapy) during follow-up. Out-of-pocket expenses will include the costs of health-related travel, over-the-counter medication etc. Volume data will be gathered with clinical report forms, available hospital information systems, and the iMTA Medical Consumption Questionnaire (iMCQ) and iMTA Productivity Cost Questionnaire (iPCQ) adjusted to the study setting. The Dutch costing guideline for health care research will be used to determine the relevant unit costs. In case of the mediastinal staging strategy however, micro-costing (general anaesthesia, surgical equipment, procedure duration, involved personnel, overhead) in participating centres will be done to estimate real unit costs. The friction costs method will be applied to derive the costs of lost productivity. After price-indexing all costs will be expressed in 2018 Euros.

Incremental cost-effectiveness ratios will be calculated with uncertainty margins based on non-parametric bias-corrected and accelerated bootstrapping. Cost-effectiveness acceptability curves will be drawn to show the probability of a strategy being cost-effective at various levels of willingness-to-pay per QALY up to 100,000 euro. In case both mediastinal staging strategies turn out clinically equivalent, the study will be performed as a cost-minimization analysis.

## Discussion

The MEDIASTrial will study whether mediastinoscopy can be omitted after negative endosonography in mediastinal staging in patients with NSCLC. Since debate exists on the additional value of mediastinoscopy, this trial will provide definite evidence on this topic [23–27]. The current literature suggests that diagnostic strategies with or without mediastinoscopy may be equivalent concerning efficacy and that abandoning mediastinoscopy appears favourable concerning morbidity and speed of diagnostic process. As a result, variety in daily practice already exists in the extent of use of mediastinoscopy throughout and within countries [7, 28, 29]. A formal comparison of cost-effectiveness and cost-utility has however never been performed and no ongoing studies comparing these two strategies have been registered in trial registers so far. Results of such a trial will have immediate impact on national and international guidelines, which are accessible to public, possibly abandoning mediastinoscopy as a commonly performed invasive procedure and diminishing variation in clinical practice.

## Additional file

Additional file 1: Model Informed Consent form. (DOCX 50 kb)

## Abbreviations

ASA: American society of anaesthesiologists
CPET: Cardiopulmonary exercise testing
CT: Computed tomography
EBUS: Endobronchial ultrasound
EBUSAT: EBUS skill and assessment tool
EORTC: European organization for research and treatment of cancer
EQ-5D-5 L: Euroqol 5 dimensions 5 Levels
ESTS: European society of thoracic surgery
EUS: Endoscopic (esophageal) ultrasonography
FDG-PET: Fluoro-deoxy-glucose positron emission tomography
IASLC: International association for the study of lung cancer
IKNL: Netherlands comprehensive cancer organisation
iMCQ: Institute medical technology assessment medical consumption questionnaire
iPCQ: Institute medical technology assessment productivity cost questionnaire
MEC: Medical ethical committee
NSCLC: Non-small cell lung cancer
QLQ-C30: Quality of life questionnaire
RATS: Robotic-assisted thoracic surgery
SUV: Standardized uptake value
TNM: Tumour, Node, Metastases
VATS: Video-assisted thoracoscopy
WHO: World health organization
WMA: World medical association
