# Supplementary material for: MEDIASTinal staging of non-small cell lung cancer by endobronchial and endoscopic ultrasonography with or without additional surgical mediastinoscopy (MEDIASTrial): study protocol of a multicenter randomised controlled trial
Source: BMC Surg. 2018 May 18;18:27. doi: 10.1186/s12893-018-0359-6 (PMC5960166; doi:10.1186/s12893-018-0359-6)
Supplement: Supplementary file 1 — Model Informed Consent form. (DOCX 50 kb) [file 12893_2018_359_MOESM1_ESM.docx]

**
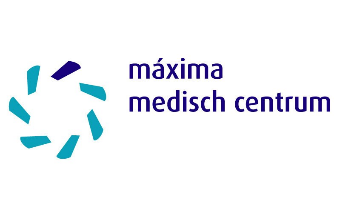
**

**Informed Consent form**

*MEDIASTinal staging of non-small cell lung cancer by endobronchial and endoscopic ultrasonography with or without additional surgical mediastinoscopy (MEDIASTrial)*

The undersigned declares to participate in the MEDIASTrial and approves the following conditions:

- A computer will determine whether you undergo a mediastinoscopy or you directly undergo an anatomical lung resection. I understand the risk of complications of both strategies.
- I have been given an opportunity to ask whatever questions I may have had and all such questions in inquiries have been answered to my satisfaction.
- I have been offered enough to time to think about participation in this trial.
- Participation in this research is entirely voluntary. It is my choice whether to participate or not. I may change my mind later and stop participating even if I agreed earlier.
- I give permission to inform my general practitioner about this participation.
- I give permission to store my medical files and tissue for 15 years, according to the Dutch law.
- I give permission to anonymously use my medical files for further research.
- I give permission to the principal investigator to inquire me for further research after completion of this trial. I decide wether or not to participate after a new informed consent procedure.
- I give permission to “Integraal Kanker Centrum Nederland (IKNL)” to forward the questionnaires using the contact information on the next page.
- I undersign this informed consent form voluntarily.

**
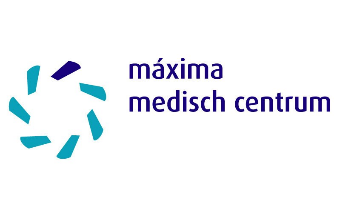
**

**Date ________________________**

**Signature participant _______________________________________________**

male  female

**Prename: ________________________________________________**

**Surname:** **________________________________________________**

**I would like to complete the questionnaires:**

Digital:

e-mail:_______________________________@__________________________

Paper:

Postal Adress: ­­­­­­­­­­­­­­­­­­­­­­­­­­­­­­­**_______________________________________________**

­­­­­­­­­­­­­­­­­­­­­­­­­­­­­­­**_______________________________________________**

­­­­­­­­­­­­­­­­­­­­­­­­­­­­­­­**_______________________________________________**

Telephone ________________________________________________

The undersigned **medical doctor** declares to have completely informed the participant about this trial according to the ‘informed consent’ procedure:

**Name ________________________________________________**

**Date ________________________________________________**

**Signature ________________________________________________**
